# A 350 kb *NEXMIF* Microdeletion Identified by Chromosomal Microarray in an Adult Patient with Jeavons Syndrome

**DOI:** 10.3390/genes17040448

**Published:** 2026-04-13

**Authors:** Mario Benvenuto, Umberto Costantino, Pietro Palumbo, Massimo Carella, Marco Castori, Giuseppe d’Orsi, Orazio Palumbo

**Affiliations:** 1 Neurological Disorders Research Unit, Fondazione IRCCS Casa Sollievo della Sofferenza, Viale Cappuccini, 71013 San Giovanni Rotondo, FG, Italy; m.benvenuto@operapadrepio.it (M.B.); m.carella@operapadrepio.it (M.C.); 2 Neurology Unit, Epilepsy Center, Fondazione IRCCS Casa Sollievo della Sofferenza, Viale Cappuccini, 71013 San Giovanni Rotondo, FG, Italy; u.costantino@operapadrepio.it; 3 Inborn Errors of Morphogenesis Research Unit, Fondazione IRCCS Casa Sollievo della Sofferenza, 71013 San Giovanni Rotondo, FG, Italy; p.palumbo@operapadrepio.it (P.P.); m.castori@operapadrepio.it (M.C.)

**Keywords:** *NEXMIF*, Jeavons syndrome, long-term follow-up, neurodevelopmental disorders

## Abstract

***Background***: Pathogenic variants in the *NEXMIF* gene have been linked to a broad neurodevelopmental phenotype, encompassing autism spectrum disorder, intellectual disability, and epilepsy. Among epileptic manifestations, Jeavons Syndrome was observed in 24% of affected females in the largest cohort of *NEXMIF*-related disorders reported to date, but long-term adult outcomes remain poorly documented. ***Methods and Results***: We report a 25-year-old Italian woman with drug-resistant Jeavons syndrome in which the combined approach of next-generation sequencing and chromosomal microarray analysis allowed us to identify, after a 13-year diagnostic odyssey, a de novo ~350 Kb microdeletion at Xq13.2q13.3 encompassing the entire *NEXMIF* coding region, with no other OMIM genes involved. To our knowledge, this is the first reported case of a patient harboring a deletion restricted to the entire coding sequence of the *NEXMIF* gene. The patient presented with moderate intellectual disability and seizure onset at age 10 years. Her epilepsy proved refractory to multiple antiseizure medications. Video-EEG/polygraphic monitoring at age 23 years confirmed epilepsy with eyelid myoclonia, demonstrating characteristic eyelid myoclonia with absences triggered by eye closure. ***Conclutions***: This case provides a detailed clinical description of an adult patient useful for genetic counseling regarding adult outcomes and prognostic expectations. Furthermore, this study underscores the diagnostic value of chromosomal microarray analysis alongside next-generation sequencing in individuals with intellectual disability and drug-resistant epilepsy, in order to expedite the diagnostic pathway and enable timelier and more appropriate patient management.

## 1. Introduction

The *NEXMIF* gene (OMIM *300524) is located at Xq13.3 and encodes the Neurite Extension and Migration Factor protein, which is highly expressed in the cerebral cortex and plays vital roles in neurite outgrowth by regulating cell–cell adhesion during early brain development [1,2,3,4]. Pathogenic *NEXMIF* variants are associated with X-linked intellectual developmental disorder 98 (XLID98, OMIM# 300912), a clinical condition characterized by delayed psychomotor development, impaired or absent speech, behavioral abnormalities, and epilepsy [5]. These variants were initially identified and characterized in hemizygous male patients with nonsyndromic X-linked intellectual disability (ID), poor or absent speech, subtle dysmorphic features, and sometimes epilepsy [6,7], while heterozygous females in these families were mostly unaffected. Subsequently, many symptomatic females carrying deleterious variants in this gene have been described, ranging from mild to severe phenotypes [5,8,9,10]. In 2021, Stamberger et al. released an extensive molecular and clinical investigation that included 87 patients with *NEXMIF*-associated encephalopathy (46 novel cases and 41 from the literature), establishing the reference standard for comparing male and female phenotypes [11]. Among the epileptic manifestations reported by females with *NEXMIF*-related disorders, epilepsy with eyelid myoclonia (EEM) emerged as a distinctive feature, occurring in 24% of affected females in their cohort.

EEM, also known as Jeavons Syndrome (JS), is a generalized epilepsy syndrome clinically characterized by eyelid myoclonia with or without absences, eye closure-induced electroencephalography paroxysms, and photosensitivity [12]. The clinical onset of this condition typically occurs during childhood, and several candidate genes are associated with this syndrome, including *SYNGAP1*, *NEXMIF*, *RORB*, *NAA10*, and *CHD2* [13].

Despite increasing recognition of *NEXMIF*-related disorders, the number of reported adult patients with pathogenic *NEXMIF* variants, particularly in association with specific epileptic syndromes such as Jeavons syndrome, remains poorly characterized. Furthermore, the mutational spectrum includes predominantly point mutations and small insertions/deletions, while large copy number variations (CNVs) remain exceptionally rare.

Here, we report the first case of a complete *NEXMIF* coding region microdeletion in a female patient with drug-resistant Jeavons syndrome and moderate intellectual disability.

## 2. Materials and Methods

### 2.1. Patient Recruitment

A blood sample from the patient and both her parents was collected at Fondazione IRCCS Casa Sollievo della Sofferenza (San Giovanni Rotondo, Italy). The family provided written informed consent to molecular testing and gave their consent to publish clinical and molecular data. The study was approved by the Casa Sollievo della Sofferenza Hospital ethics committee (protocol no. 90/CE/2024) and is in accordance with the Declaration of Helsinki (1984) and subsequent versions.

Genomic DNA was extracted from peripheral blood leukocytes using the automated Bio Robot EZ1 (Quiagen, Solna, Sweden) following the manufacturer’s instructions. DNA concentration and purity were assessed spectrophotometrically using the Nanodrop 2000C spectrophotometer (Thermo Fisher Scientific, Waltham, MA, USA).

### 2.2. Genetic Analysis

In order to expedite the diagnostic procedure, on the proband’s DNA, we performed Gene panel sequencing and Chromosomal microarray analysis simultaneously.

Gene panel sequencing was performed using custom SureSelect gene panels (Agilent Technologies, Boulder, CA, USA) designed to selectively capture 162 and 294 known genes associated with syndromic and non-syndromic forms of epilepsy and neurodevelopmental disorders (Supplementary Materials and Methods), respectively. Libraries were prepared using the SureSelect enrichment kit (Agilent Technologies, Boulder, CO, USA) following the manufacturer’s instructions.

Subsequently, the targeted fragments obtained were sequenced on a NextSeq 500 sequencer (Illumina, San Diego, CA, USA) using a NextSeq 500 mid-output kit V2.5 (300-cycle flow cell), and identified variants were prioritized as previously described [14]. The clinical significance of the identified putative variants was assessed according to the American College of Medical Genetics and Genomics (ACMG) guidelines [15].

Chromosomal microarray analysis (CMA) was performed using the CytoScan HD Array (Thermo Fisher Scientific, Waltham, MA, USA) according to the manufacturer’s protocol, while data analysis was performed by processing Raw data (CEL files) with Chromosome Analysis Suite (ChAS) software version 4.3 (Thermo Fisher Scientific) as previously described [16]. In addition to the American College of Medical Genetics (ACMG) and Clinical Genome Resource (ClinGen) guidelines [17], the clinical significance of each identified CNV was established by comparing the chromosomal alterations found in the patient with those from public databases such as the Database of Genomic Variants (DGV) (https://dgv.tcag.ca/dgv/app/home, accessed on 2 February 2026), ClinVar (https://www.ncbi.nlm.nih.gov/clinvar/, accessed on 2 February 2026), DECIPHER (https://www.deciphergenomics.org, accessed on 2 February 2026), SFARI (Simon’s Foundation Autism Research Initiative, https://gene.sfari.org, accessed on 2 February 2026), and OMIM (Online Mendelian Inheritance in Man database, https://www.omim.org/, accessed on 2 February 2026). Genomic coordinates, genes impacted by CNV, and disease associations were determined from the University of California Santa Cruz (UCSC) Genome Browser (GRCh38) (https://genome.ucsc.edu, accessed on 2 February 2026).

## 3. Results

### 3.1. Clinical Description

The patient is a 25-year-old Italian woman, firstborn of two children from unrelated and healthy parents. Family history for neurological disorders was unremarkable. Perinatal distress was noted, including maternal hypertensive crises and an urgent Cesarean section at term (39 weeks of gestation) due to fetal distress. Birth weight was 2200 gr (<3rd percentile), and birth length was 46 cm (10–25th percentile). The neonatal period following the perinatal distress was unremarkable, with no recorded enduring complications. Since early childhood, the patient has shown a psychomotor developmental delay and moderate intellectual disability, requiring educational support throughout primary school. Despite these challenges, she successfully completed her secondary education, suggesting that her practical skills and ability to perform structured tasks were relatively intact compared to her overall cognitive abilities.

**Figure 1:**
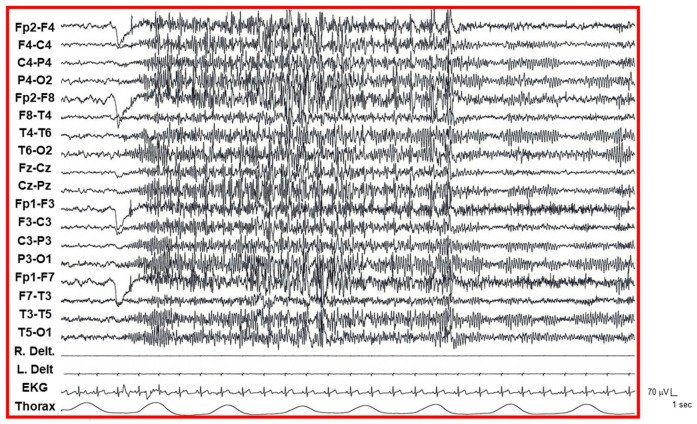
EEG/polygraphic recording with bilateral deltoid EMG (right and left channels), single-lead ECG, and thoracic respiration monitoring. Upon eye closure, after the initial muscular artifact from eyelid closure, generalized paroxysmal fast activity (GPFA) emerges, persisting for approximately 15 s before gradually resolving. As the GPFA attenuates, the bilateral posterior alpha rhythm becomes progressively more apparent over the posterior derivations, having been initially obscured by the overlying GPFA. Note the preservation of normal posterior dominant rhythm throughout the recording. Calibration bars (70 μV and 1 s) are provided in the bottom right corner.

At age 10 years, she developed daily seizures characterized by brief absences associated with generalized spike-and-polyspike-and-slow-wave discharges on electroencephalography (EEG). These episodes frequently evolved into prolonged non-convulsive status epilepticus and proved refractory to multiple antiseizure medications, including valproic acid (VPA), ethosuximide (ETS), lamotrigine (LTG), topiramate (TPM), and perampanel (PER). At least two generalized tonic–clonic seizures occurred at ages 15 and 18 years. Prolonged video-EEG/polygraphic monitoring at age 23 years revealed brief and prolonged bursts (5–10 s) of low-amplitude, diffuse, fast spike-and-polyspike-and-slow-wave discharges. These abnormalities were accentuated by eye closure and clinically associated with ocular deviation (Figure 1). The electroclinical pattern was consistent with epilepsy with eyelid myoclonia (formerly Jeavons syndrome).

Brain magnetic resonance imaging (MRI) was unremarkable.

At 25 years old, she underwent her last physical examination, which revealed a height of 152 cm (3rd–5th percentile), a weight of 82 kg, a body mass index (BMI) of 35.5 kg/m^2^ (obesity class II), and a head circumference of 53 cm (50th percentile). Subtle dysmorphic features, including mild left eyelid ptosis and bilateral pes planus (flat feet), were noted.

### 3.2. Genetic Findings

**Figure 2:**
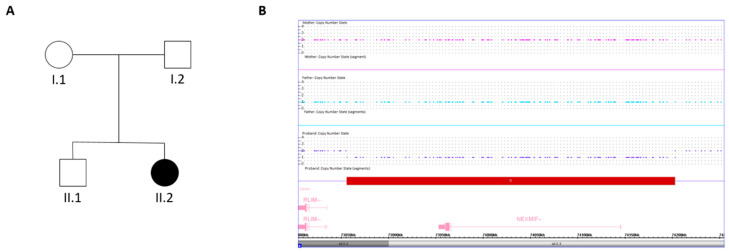
(**A**) Pedigree of the family. Filled and unfilled circles/squares represent affected and unaffected individuals, respectively. (**B**) Results of SNP-array analysis in the analyzed individuals. Copy number state of each probe is drawn along chromosome X (UCSC Genome Browser, build GRCh38). The upper panel represents the copy number state of the mother (I.1), the middle panel represents the copy number state of the father (I.2), and the lower panel that of the proband (II.2). Values of the Y-axis indicate the inferred copy number according to the probes intensities. Red bar indicates the Xq13.2q13.3 microdeletions identified in the patient.

No pathogenic or likely pathogenic sequence variants were detected by gene panels sequencing, while CMA revealed a heterozygous microdeletion at locus Xq13.2q13.3, spanning approximately 350 kb. The deletion encompassed only the entire coding region of the *NEXMIF* gene and was covered by 427 SNP array probes. The proximal breakpoint (centromeric) was located between the last present probe C-5WYQT (74,634,506 bp) and the first deleted probe S-4AECE (74,635,750 bp), while the distal breakpoint (telomeric) was located between the last deleted probe C-5REDO (74,982,780 bp) and the first present probe C-6TPYO (74,982,820 bp). Subsequently, the parental CMA showed a normal copy number state in both parents, suggesting the de novo occurrence of the microdeletion (Figure 2).

The highlighted region did not show any benign copy number variants in the DGV database. Furthermore, neither the ClinVar nor the DECIPHER databases contained any annotated patients with similar rearrangements. The molecular karyotype of the patient, according to the International System for Human Cytogenetic Nomenclature (ISCN 2020), is arr[GRCh38] Xq13.2q13.3(74635750_74982780)x1 dn.

Following ACMG/ClinGen guidelines for CNV interpretation, this microdeletion was classified as “pathogenic” based on the complete overlap of an established haploinsufficiency (HI)/loss of function (LOF)—sensitive gene/genomic region (total score: +1.00, 2A), associated with a syndromic form of X-linked neurodevelopmental disorder. We did not perform X chromosome inactivation (XCI) analysis in blood, as XCI patterns exhibit significant heterogeneity across tissues, and the skewing observed in blood samples may not reflect the pattern in clinically relevant tissues such as the brain or other organs [11].

## 4. Discussion

In this study we report the clinical and molecular characterization of a 25-year-old Italian woman harboring a de novo ~350 Kb interstitial microdeletion on Xq13.2q13.3 chromosome region. This microdeletion encompasses the complete coding region of the *NEXMIF* gene. The patient presented with a complex neurodevelopmental phenotype characterized by drug-resistant Jeavons syndrome (JS), moderate intellectual disability, and obesity.

*NEXMIF*-related disorders (OMIM # 300912) are X-linked neurodevelopmental conditions caused by pathogenic variants in the *NEXMIF*, leading to intellectual disability, autism spectrum disorder, and epilepsy. The condition shows a pronounced sex influence, with males typically having more severe developmental impairment and exhibiting more severe intellectual disability and behavioral abnormalities, consistent with X-linked inheritance, whereas females have a disproportionately high seizure rate [11]. Jeavons syndrome has been identified as a distinctive feature in 24% of affected females in the largest cohort of *NEXMIF*-related disorders reported to date [11]. This case represents long-term longitudinal documented clinical follow-ups, carrying the pathogenic *NEXMIF* deletion associated with JS.

The longitudinal observation reveals three clinical stages in our patient: (i) she showed seizure onset at 10 years with rapid evolution of severe pharmacoresistance; (ii) adolescent progression was marked by persistent absence seizures, status epilepticus, and two generalized tonic–clonic seizures; (iii) adult progression was characterized by frequent, drug-resistant absence status epilepticus but was stable from a cognitive perspective.

Seizure onset in Jeavons syndrome typically occurs in childhood between ages 2–14 years, with most cases presenting between ages 6–8 years [18]. Our patient had seizure onset at 10 years and subsequently developed severe drug-resistant epilepsy, resistant to five pharmacological treatments, including VPA, ETS, LTG, TPM, and PER. Valproic acid, ethosuximide, and lamotrigine are three of the four most effective treatments identified in JS [18], underscoring the severe pharmacoresistance in our case. She successfully completed secondary education despite daily seizures and moderate intellectual disability, suggesting that educational achievement may be preserved in selected NEXMIF-related cases despite significant seizure burden. This outcome extends observations from JS, in which 23% of patients have intellectual disability [19], but does not preclude functional or educational achievements. The development of obesity during adolescence is consistent with Langley et al. (2022), who suggested that obesity may be an underestimated feature of *NEXMIF*-related disorders [7].

Video-EEG/polygraphic monitoring only established the diagnosis of Jeavons syndrome at age 23, highlighting the diagnostic challenges inherent to these disorders. Finally, these long-term remarks offer valuable, useful prognostic insights for counseling families and underscore the need for systematic longitudinal studies to better understand natural history patterns in *NEXMIF*-related disorders.

From a molecular point of view, this case represents the first report describing a complete *NEXMIF* coding region deletion in a female with Jeavons syndrome. The mutational spectrum of *NEXMIF*-related disorders can be defined by loss-of-function point mutations and small indels, whereas large copy number variations are uncommon. In 2021, Stamberger et al. reported a female carrying a microdeletion of 77 kb that covers a part of the *NEXMIF* gene [11]. The microdeletion identified in our patient does not encompass any other OMIM genes, providing a direct genotype-phenotype correlation. A query in the ClinVar and DECIPHER databases (February, 2026) confirmed that there are no previously reported cases with microdeletion that involves the entire *NEXMIF* coding region with no other contiguous OMIM genes. This finding expands the mutational landscape and provides further molecular evidence supporting haploinsufficiency as the primary pathogenic mechanism in this syndrome.

To acquire a mechanistic understanding of epileptogenesis, it is essential to understand the biological function of the NEXMIF protein. *NEXMIF* is an X-linked gene that encodes a nuclear protein highly expressed in the cerebral cortex. It is crucial in neuronal morphogenesis, migration, and synapse formation [1,2,4]. Loss-of-function variants lead to abnormal dendritic arborization and decreased synaptic density [1,4], which may disrupt excitatory/inhibitory balance and predispose individuals to hyperexcitability. The high prevalence of generalized epilepsy with absence features in *NEXMIF* patients [11] suggests the involvement of circuits generating spike-wave discharges.

In addition, a key factor influencing the phenotype of heterozygous female carriers of X-linked variants is X-chromosome inactivation (XCI). Although XCI is typically random, resulting in cellular mosaicism, it is reasonable to assume that NEXMIF deficiency likely operates through a cellular interference mechanism. Similarly to *PCDH19*—another gene involved in cell–cell adhesion and neuronal migration—the presence of cellular mosaicism in the brain may generate network instability, potentially predisposing individuals to epileptogenesis more potently than a uniform loss of function. Recent functional studies and animal models support this hypothesis [20]. Furthermore, other studies have shown that affected female carriers do not exhibit a direct correlation between phenotype and XCI status, suggesting that simply analyzing skewing in peripheral blood, which might not reflect the patterns in brain tissue, does not fully explain phenotypic expression [11]. Consequently, this cellular interference mechanism could offer an explanation for the severe drug resistance observed in our patient, as the mixed neuronal populations may create complex network instability.

## 5. Conclusions

This report describes an adult case of refractory Jeavons Syndrome associated with a complete *NEXMIF* coding sequence microdeletion, identified after 13 years. Our findings highlight the importance of multi-modal genetic testing in complex cases and the value of long-term follow-up in adults. Characterizing the natural history of *NEXMIF*-related disorders is crucial for improving lifelong clinical management and genetic counseling for affected families.

## Data Availability

The data that support the findings of this study are available from the corresponding author upon reasonable request.

## Supplementary Materials

The following supporting information can be downloaded at: https://www.mdpi.com/article/10.3390/genes17040448/s1, Supplementary Materials and Methods.
