# Supplementary material for: A 350 kb NEXMIF Microdeletion Identified by Chromosomal Microarray in an Adult Patient with Jeavons Syndrome
Source: Genes (Basel). 2026 Apr 13;17(4):448. doi: 10.3390/genes17040448 (PMC13115607; doi:10.3390/genes17040448)
Supplement: Supplementary file 1 [file genes-17-00448-s001.zip › genes-4245995-supplementary.pdf]

A 350 kb NEXMIF Microdeletion Identified by Chromosomal Microarray in an Adult Patient with Jeavons Syndrome

Mario Benvenuto[1], Umberto Costantino[2], Pietro Palumbo[3], Massimo Carella[1], Marco Castori[3], Giuseppe d'Orsi[2]*, and Orazio Palumbo[3]*

[1]Neurological Disorders Research Unit, Fondazione IRCCS Casa Sollievo della Sofferenza, Viale Cappuccini, San Giovanni Rotondo, FG, Italy

[2]Neurology Unit - Epilepsy Center, Fondazione IRCCS Casa Sollievo della Sofferenza, Viale Cappuccini, San Giovanni Rotondo, FG, Italy

[3]Inborn Errors of Morphogenesis Research Unit, Fondazione IRCCS Casa Sollievo della Sofferenza, San Giovanni Rotondo, FG, Italy

* Correspondence: g.dorsi@operapadrepio.it (G.d.); o.palumbo@operapadrepio.it (O.P.);

Tel.: +39-06-446288 (G.d.); +39-06-446345 (O.P.)

Supplementary Materials and Methods

Neurodevelopmental disorders gene panel:
ACTB (OMIM *102630), *ACTG1* (OMIM *102560), *ACY1* (OMIM *104620), *ADNP* (OMIM *611386), *ADSL* (OMIM *608222), *AFF2* (OMIM *300806), *AHDC1* (OMIM *615790), *AHI1* (OMIM *608894), *ALDH5A1* (OMIM *610045), *ANKRD11* (OMIM *611192), *ANKRD17* (OMIM *615929), *AP1S2* (OMIM *300629), *APC2* (OMIM *612034), *ARFGEF2* (OMIM *605371), *ARHGEF9* (OMIM *300429), *ARID1A* (OMIM *603024), *ARID1B* (OMIM *614556), *ARID2* (OMIM *609539), *ARX* (OMIM *300382), *ASH1L* (OMIM *607999), *ASPM* (OMIM *605481), *ASXL1* (OMIM *612990), *ASXL2* (OMIM *612991), *ASXL3* (OMIM *615115), *ATAD3A* (OMIM *612316), *ATP1A1* (OMIM *182310), *ATP1A3* (OMIM *182350), *ATRX* (OMIM *300032), *AUTS2* (OMIM *607270), *BBS4* (OMIM *600374), *BCL11A* (OMIM *606557), *BPTF* (OMIM *601819), *BRAF* (OMIM *164757), *BRPF1* (OMIM *602410), *BRWD3* (OMIM *300553), *C12ORF57* (OMIM *615140), *CACNA1A* (OMIM *601011), *CAMK2A* (OMIM *114078), *CAMK2B* (OMIM *607707), *CASK* (OMIM *300172), *CC2D1A* (OMIM *610055), *CDC42BPB* (OMIM *614062), *CDK13* (OMIM *603309), *CDKL5* (OMIM *300203), *CDKN1C* (OMIM *600856), *CELF2* (OMIM *602538), *CEP290* (OMIM *610142), *CHAMP1* (OMIM *616327), *CHD2* (OMIM *602119), *CHD7* (OMIM *608892), *CHD8* (OMIM *610528), *CIC* (OMIM *612082), *CLCN4* (OMIM *302910), *CLTC* (OMIM *118955), *CNKSR2* (OMIM *300724), *CNOT1* (OMIM *604917), *CNOT3* (OMIM *604910), *CREBBP* (OMIM *600140), *CSNK2A1* (OMIM *115440), *CSNK2B* (OMIM *115441), *CTCF* (OMIM *604167), *CTNNB1* (OMIM *116806), *CUL4B* (OMIM *300304), *CUX2* (OMIM *610648), *DDHD2* (OMIM *615003), *DDX3X* (OMIM *300160), *DEAF1* (OMIM *602635), *DLG3* (OMIM *300189), *DLG4* (OMIM *602887), *DLL1* (OMIM *606582), *DMD* (OMIM *300377), *DNMT3A* (OMIM *602769), *DPF2* (OMIM *601671), *DYNC1H1* (OMIM *600112), *DYRK1A* (OMIM *600855), *EBF3* (OMIM *607407), *EED* (OMIM *605984), *EEF1A2* (OMIM *602959), *EHMT1* (OMIM *607001), *EP300* (OMIM *602700), *ERLIN2* (OMIM *611605), *EZH2* (OMIM *601573), *FBXO11* (OMIM *607871), *FGD1* (OMIM *300546), *FMR1* (OMIM *309550), *FOXG1* (OMIM *164874), *FOXP1* (OMIM *605515), *FOXP2* (OMIM *605317), *FTO* (OMIM *610966), *GABBR2* (OMIM *607340), *GABRB2* (OMIM *600232), *GABRB3* (OMIM *137192), *GATAD2B* (OMIM *614998), *GDI1* (OMIM *300104), *GNB1* (OMIM *139380), *GPC3* (OMIM *300037), *GRIA3* (OMIM *305915), *GRIK2* (OMIM *138244), *GRIN1* (OMIM *138249), *GRIN2A* (OMIM *138253), *GRIN2B* (OMIM *138252), *HCN1* (OMIM *602780), *HDAC4* (OMIM *605314), *HDAC8* (OMIM *300269), *HERC1* (OMIM *605109), *HERC2* (OMIM *605837), *HIVEP2* (OMIM *143054), *HNRNPK* (OMIM *600712), *HOXA1* (OMIM *142955), *HPRT1* (OMIM *308000), *HRAS* (OMIM *190020), *HUWE1* (OMIM *300697), *IL1RAPL1* (OMIM *300206), *INTS1* (OMIM *611345), *IQSEC2* (OMIM *300522), *IRF2BPL* (OMIM *611720), *ITPR1* (OMIM *147265), *KANSL1* (OMIM *612452), *KAT6A* (OMIM *601408), *KAT6B* (OMIM *605880), *KCNA2* (OMIM *176262), *KCNB1* (OMIM *600397), *KCNJ10* (OMIM *602208), *KCNMA1* (OMIM *600150), *KCNQ2* (OMIM *602235), *KDM3B* (OMIM *609373), *KDM4B* (OMIM *609765), *KDM5B* (OMIM *605393), *KDM5C* (OMIM *314690), *KDM6A* (OMIM *300128), *KDM6B* (OMIM *611577), *KIF1A* (OMIM *601255), *KMT2A* (OMIM *159555), *KMT2B* (OMIM *606834), *KMT2C* (OMIM *606833), *KMT2D* (OMIM *602113), *KMT2E* (OMIM *608444), *KPTN* (OMIM *615620), *L1CAM* (OMIM *308840), *LARP7* (OMIM *612026), *LINS1* (OMIM *610350), *LRP2* (OMIM *600073), *LZTR1* (OMIM *600574), *MAGEL2* (OMIM *605283), *MAN1B1* (OMIM *604346), *MAOA* (OMIM *309850), *MBD5* (OMIM *611472), *MBOAT7* (OMIM *606048), *MBTPS2* (OMIM *300294), *MCPH1* (OMIM *607117), *MECP2* (OMIM *300005), *MED12* (OMIM *300188), *MED13* (OMIM *603808), *MED13L* (OMIM *608771), *MED23* (OMIM *605042), *MEF2C* (OMIM *600662), *MEIS2* (OMIM *601740), *METTL23* (OMIM *615262), *MID1* (OMIM *300552), *MSL3* (OMIM *300609), *MTOR* (OMIM *601231), *MYO5A* (OMIM *160777), *MYT1L* (OMIM *613084), *NAA15* (OMIM *608000), *NACC1* (OMIM *610672), *NBEA* (OMIM *604889), *NEXMIF* (OMIM *300524), *NF1* (OMIM *613113), *NFIA* (OMIM *600727), *NFIX* (OMIM *164005), *NHS* (OMIM *300457), *NIPBL* (OMIM *608667), *NR2F1* (OMIM *132890), *NRXN1* (OMIM *600565), *NSD1* (OMIM *606681), *NSD2* (OMIM *602952), *NSUN2* (OMIM *610916), *NTNG2* (OMIM *618689), *NTRK2* (OMIM *600456), *OFD1* (OMIM *300170), *OPHN1* (OMIM *300127), *PAH* (OMIM *612349),

PAK3 (OMIM *300142), *PCDH19* (OMIM *300460), *PGAP1* (OMIM *611655), *PHF21A* (OMIM *608325), *PHF6* (OMIM *300414), *PHF8* (OMIM *300560), *PHIP* (OMIM *612870), *PIGG* (OMIM *616918), *PIK3CA* (OMIM *171834), *PLP1* (OMIM *300401), *POGZ* (OMIM *614787), *POLR3A* (OMIM *614258), *POU3F3* (OMIM *602480), *PPM1D* (OMIM *605100), *PPP2R5D* (OMIM *601646), *PPP3CA* (OMIM *114105), *PQBP1* (OMIM *300463), *PRR12* (OMIM *616633), *PSMD12* (OMIM *604450), *PTCHD1* (OMIM *300828), *PTEN* (OMIM *601728), *PTPN11* (OMIM *176876), *PURA* (OMIM *600473), *QRICH1* (OMIM *617387), *RAB39B* (OMIM *300774), *RAC1* (OMIM *602048), *RAD21* (OMIM *606462), *RAI1* (OMIM *607642), *RBM10* (OMIM *300080), *RELN* (OMIM *600514), *RERE* (OMIM *605226), *RLIM* (OMIM *300379), *RORA* (OMIM *600825), *RPS6KA3* (OMIM *300075), *SATB2* (OMIM *608148), *SCN1A* (OMIM *182389), *SCN2A* (OMIM *182390), *SCN8A* (OMIM *600702), *SEMA3E* (OMIM *608166), *SET* (OMIM *600960), *SETBP1* (OMIM *611060), *SETD1A* (OMIM *611052), *SETD1B* (OMIM *611055), *SETD2* (OMIM *612778), *SETD5* (OMIM *615743), *SHANK2* (OMIM *603290), *SHANK3* (OMIM *606230), *SIN3A* (OMIM *607776), *SLC16A2* (OMIM *300095), *SLC6A1* (OMIM *137165), *SLC6A8* (OMIM *300036), *SLC9A6* (OMIM *300231), *SMARCA2* (OMIM *600014), *SMARCA4* (OMIM *603254), *SMARCB1* (OMIM *601607), *SMARCC2* (OMIM *601734), *SMARCE1* (OMIM *603111), *SMC1A* (OMIM *300040), *SMC3* (OMIM *606062), *SMS* (OMIM *300105), *SNX14* (OMIM *616105), *SON* (OMIM *182465), *SOX11* (OMIM *600898), *SOX4* (OMIM *184430), *SOX5* (OMIM *604975), *SPTBN1* (OMIM *182790), *SRCAP* (OMIM *611421), *STAG1* (OMIM *604358), *STAG2* (OMIM *300826), *STXBP1* (OMIM *602926), *SUZ12* (OMIM *606245), *SYN1* (OMIM *313440), *SYNGAP1* (OMIM *603384), *TAF1* (OMIM *313650), *TANC2* (OMIM *615047), *TAOK1* (OMIM *610266), *TBCK* (OMIM *616899), *TBL1XR1* (OMIM *608628), *TBR1* (OMIM *604616), *TBX1* (OMIM *602054), *TCF20* (OMIM *603107), *TCF4* (OMIM *602272), *TLK2* (OMIM *608439), *TNRC6B* (OMIM *610740), *TRAF7* (OMIM *606692), *TRAPPC9* (OMIM *611966), *TRIO* (OMIM *601893), *TRIP12* (OMIM *604506), *TSC1* (OMIM *605284), *TSC2* (OMIM *191092), *UBE2A* (OMIM *312180), *UBE3A* (OMIM *601623), *UNC80* (OMIM *612636), *UPF3B* (OMIM *300298), *USP7* (OMIM *602519), *USP9X* (OMIM *300072), *VAMP2* (OMIM *185881), *VARS1* (OMIM *192150), *VPS13B* (OMIM *607817), *WAC* (OMIM *615049), *WASF1* (OMIM *605035), *WDFY3* (OMIM *617485), *WDR26* (OMIM *617424), *WWOX* (OMIM *605131), *YY1* (OMIM *600013), *ZBTB18* (OMIM *608433), *ZBTB20* (OMIM *606025), *ZDHHC9* (OMIM *300646), *ZEB2* (OMIM *605802), *ZFYVE26* (OMIM *612012), *ZMYND11* (OMIM *608668), *ZNF462* (OMIM *617371).

Epilepsy gene panel:
ABCB1 (OMIM *171050), *ABCC2* (OMIM *601107), *ADGRV1* (OMIM *602851), *ADSL* (OMIM *608222), *AFG2A* (OMIM *613940), *ALDH5A1* (OMIM *610045), *ALDH7A1* (OMIM *107323), *ALG13* (OMIM *300776), *AP2M1* (OMIM *601024), *ARHGEF9* (OMIM *300429), *ARX* (OMIM *300382), *ATP1A2* (OMIM *182340), *ATP1A3* (OMIM *182350), *ATP6V1A* (OMIM *607027), *BRAT1* (OMIM *614506), *CACNA1A* (OMIM *601011), *CACNA1D* (OMIM *114206), *CACNA1E* (OMIM *601013), *CACNA1H* (OMIM *607904), *CACNB4* (OMIM *601949), *CASK* (OMIM *300172), *CDK19* (OMIM *614720), *CDKL5* (OMIM *300203), *CHD2* (OMIM *602119), *CHRNA2* (OMIM *118502), *CHRNA4* (OMIM *118504), *CHRNA7* (OMIM *118511), *CHRNB2* (OMIM *118507), *CLCN2* (OMIM *600570), *CLN3* (OMIM *607042), *CLN5* (OMIM *608102), *CLN6* (OMIM *606725), *CLN8* (OMIM *607837), *CLTC* (OMIM *118955), *CNKSR2* (OMIM *300724), *CNTNAP2* (OMIM *604569), *CPA6* (OMIM *609562), *CSNK2B* (OMIM *115441), *CSTB* (OMIM *601145), *CTSD* (OMIM *116840), *CTSF* (OMIM *603539), *CUL4B* (OMIM *300304), *CUX2* (OMIM *610648), *CYFIP2* (OMIM *606323), *CYP2C9* (OMIM *601130), *DDX3X* (OMIM *300160), *DENND5A* (OMIM *617278), *DEPDC5* (OMIM *614191), *DHDDS* (OMIM *608172), *DLL1* (OMIM *606582), *DNAJC5* (OMIM *611203), *DNM1* (OMIM *602377), *DOCK7* (OMIM *615730), *DOLK* (OMIM *610746), *DYNC1H1* (OMIM *600112), *EEF1A2* (OMIM *602959), *EFHC1* (OMIM *608815), *EPM2A* (OMIM *607566), *FGF12* (OMIM *601513), *FGF13* (OMIM *300070), *FOLR1* (OMIM *136430), *FOXG1* (OMIM *164874), *FRRS1L* (OMIM *604574), *GABBR2* (OMIM *607340), *GABRA1* (OMIM *137160), *GABRA2* (OMIM *137140), *GABRA5* (OMIM *137142), *GABRB2* (OMIM *600232), *GABRB3* (OMIM

*137192), *GABRD* (OMIM *137163), *GABRG2* (OMIM *137164), *GAMT* (OMIM *601240), *GATM* (OMIM *602360), *GLDC* (OMIM *238300), *GNAO1* (OMIM *139311), *GOSR2* (OMIM *604027), *GRIN1* (OMIM *138249), *GRIN2A* (OMIM *138253), *GRIN2B* (OMIM *138252), *GRIN2D* (OMIM *602717), *HCN1* (OMIM *602780), *HCN2* (OMIM *602781), *HLA-B* (OMIM *142830), *HNRNPU* (OMIM *602869), *IER3IP1* (OMIM *609382), *IQSEC2* (OMIM *300522), *IRF2BPL* (OMIM *611720), *KCNA1* (OMIM *176260), *KCNA2* (OMIM *176262), *KCNB1* (OMIM *600397), *KCNC1* (OMIM *176258), *KCNJ10* (OMIM *602208), *KCNMA1* (OMIM *600150), *KCNQ2* (OMIM *602235), *KCNQ3* (OMIM *602232), *KCNT1* (OMIM *608167), *KCNT2* (OMIM *610044), *KCTD7* (OMIM *611725), *LGI1* (OMIM *604619), *LIAS* (OMIM *607031), *MECP2* (OMIM *300005), *MEF2C* (OMIM *600662), *MFSD8* (OMIM *611124), *NECAP1* (OMIM *611623), *NEXMIF* (OMIM *300524), *NHLRC1* (OMIM *608072), *NPRL2* (OMIM *607072), *NPRL3* (OMIM *600928), *NRXN1* (OMIM *600565), *NTRK2* (OMIM *600456), *PCDH19* (OMIM *300460), *PIGA* (OMIM *311770), *PIGG* (OMIM *616918), *PLCB1* (OMIM *607120), *PNKP* (OMIM *605610), *PNPO* (OMIM *603287), *POLG* (OMIM *174763), *PPT1* (OMIM *600722), *PRICKLE1* (OMIM *608500), *PRICKLE2* (OMIM *608501), *PRRT2* (OMIM *614386), *PURA* (OMIM *600473), *QARS1* (OMIM *603727), *RELN* (OMIM *600514), *RORB* (OMIM *601972), *SCARB2* (OMIM *602257), *SCN1A* (OMIM *182389), *SCN1B* (OMIM *600235), *SCN2A* (OMIM *182390), *SCN3A* (OMIM *182391), *SCN8A* (OMIM *600702), *SEMA6B* (OMIM *608873), *SERPINI1* (OMIM *602445), *SLC12A5* (OMIM *606726), *SLC13A5* (OMIM *608305), *SLC19A3* (OMIM *606152), *SLC25A22* (OMIM *609302), *SLC2A1* (OMIM *138140), *SLC35A2* (OMIM *314375), *SLC6A1* (OMIM *137165), *SLC6A8* (OMIM *300036), *SLC9A6* (OMIM *300231), *SPTAN1* (OMIM *182810), *ST3GAL3* (OMIM *606494), *STX1B* (OMIM *601485), *STXBP1* (OMIM *602926), *SYN1* (OMIM *313440), *SYNGAP1* (OMIM *603384), *SYNJ1* (OMIM *604297), *SZT2* (OMIM *615463), *TBC1D24* (OMIM *613577), *TCF4* (OMIM *602272), *TPP1* (OMIM *607998), *TRAK1* (OMIM *608112), *TRPM6* (OMIM *607009), *TSC1* (OMIM *605284), *TSC2* (OMIM *191092), *UBE3A* (OMIM *601623), *WDR45* (OMIM *300526), *WWOX* (OMIM *605131), *YWHAG* (OMIM *605356), *ZEB2* (OMIM *605802).
